# Generation of a macroscopic entangled coherent state using quantum memories in circuit QED

**DOI:** 10.1038/srep32004

**Published:** 2016-08-26

**Authors:** Tong Liu, Qi-Ping Su, Shao-Jie Xiong, Jin-Ming Liu, Chui-Ping Yang, Franco Nori

**Affiliations:** 1 Department of Physics, Hangzhou Normal University, Hangzhou, Zhejiang 310036, China; 2 State Key Laboratory of Precision Spectroscopy, Department of Physics, East China Normal University, Shanghai 200062, China; 3 CEMS, RIKEN, Saitama 351-0198, Japan; 4 Department of Physics, The University of Michigan, Ann Arbor, Michigan 48109-1040, USA

## Abstract

*W*-type entangled states can be used as quantum channels for, e.g., quantum teleportation, quantum dense coding, and quantum key distribution. In this work, we propose a way to generate a macroscopic *W*-type entangled coherent state using quantum memories in circuit QED. The memories considered here are nitrogen-vacancy center ensembles (NVEs), each located in a different cavity. This proposal does not require initially preparing each NVE in a coherent state instead of a ground state, which should significantly reduce its experimental difficulty. For most of the operation time, each cavity remains in a vacuum state, thus decoherence caused by the cavity decay and the unwanted inter-cavity crosstalk are greatly suppressed. Moreover, only one external-cavity coupler qubit is needed, which simplifies the circuit.

Unlike bipartite systems, it has been proven that there exist two inequivalent classes of multipartite entangled states, such as GHZ states^1^ and *W* states^2^, which cannot be converted to each other by local operations and classical communications. Relative to the tripartite entangled states, GHZ states are fragile: if any one qubit is traced out, the remaining bipartite states are separable states. However, *W* states are robust against qubit loss and qubit-flip noise because they maintain bipartite entanglement. *W* states are important for quantum communications. For example, *W* states can be used as quantum channels for quantum teleportation^3^, quantum dense coding^4^, and quantum key distribution^5^.

Over the past years, a number of theoretical ideas have been proposed for creating a *discrete-variable W*-class entangled state 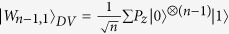 of qubits (i.e., *two-state* particles or *two-level* quantum systems)^6^,^7^,^8^,^9^,^10^,^11^,^12^,^13^, where *P*_*z*_ is the symmetry permutation operator for the qubits (1, 2 ···*n*), and 
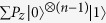
 denotes the totally-symmetric state in which (*n* − 1) qubits out of a total of *n* qubits are in the state |0〉, while the remaining qubit is in the state |1〉. As an example, consider a three-qubit case (i.e., *n* = 3), for which the *W* state is 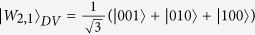. Experimentally, the discrete-variable *W* states |*W*_*n*−1,1_〉_*DV*_ have been created with up to eight trapped ions^14^, four optical modes^15^, three superconducting phase qubits coupled capacitively^16^, atomic ensembles in four quantum memories^17^, and two superconducting phase qubits plus a resonant cavity^18^.

On the other hand, there is much interest in *entangled coherent states* (ECSs)^19^,^20^,^21^,^22^,^23^,^24^,^25^,^26^,^27^,^28^. In this work we focus on a macroscopic *W*-type ECS (i.e., *continuous-variable W* state), described by


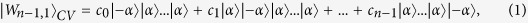


where 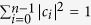, with *c*_*i*_ ≠ 0 (*i* = 0, 1,..., *n* − 1), 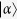 (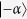) is a coherent state, *α* is a complex number, and 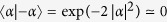, when |*α*| is large enough. The *W* state (1) is of fundamental interest in quantum mechanics and plays an important role in quantum information processing (QIP) and quantum communications. For instance, the *W* state (1) can be used to test quantum nonlocality without inequality^29^,^30^ and the violation of the Bell inequalities because such state is greater than that for any states involving two spin-1/2 particles^30^,^31^. In addition, ref. 32 has shown that there exists a quantum information protocol which is not suitable for GHZ-type ECSs but can only be accomplished with the *W* state (1). Moreover, the *W* state (1) is a necessary resource for remote symmetric entanglement^32^, which allows two distant parties to share a symmetric entangled state. For the past years, theoretical methods have been proposed for generating the *W* state (1) in some physical systems^33^,^34^,^35^,^36^,^37^. Refs 32, 33, 34 have proposed how to generate the *W* state (1) of three/four modes with linear optical devices, and refs 36 and 37 have discussed how to create the *W* state (1) of three-cavity fields based on cavity QED. However, in these schemes, the *W* ECSs were prepared with photons or cavity fields, and thus decoherence may pose a problem due to photon loss or cavity-field decay.

Hybrid quantum systems, composed of superconducting qubits, nitrogen-vacancy centers (NVCs), nitrogen-vacancy center ensembles (NVEs), or/and superconducting microwave resonators/cavities, have attracted tremendous attention^38^,^39^,^40^,^41^. Recently, much progress has been made in this field. For instance, coherent coupling between a superconducting flux/transmon qubit and an NVE^42^,^43^ or between an NVC/NVE and a superconducting resonator^44^,^45^ has been experimentally demonstrated. Moreover, based on the hybrid systems, various quantum operations, such as entanglement preparation, quantum logic gates, and information transfer, have been investigated in theory^40^,^46^,^47^,^48^,^49^ and demonstrated in experiment^42^,^50^,^51^.

Inspired by previous works and the long decoherence time of NVEs, we here consider a hybrid system composed of one-dimensional transmission line resonators (TLRs) each hosting an NVE and a qubit and connected to a coupler qubit *A* [Figs 1(a) and 2]. We then propose a way to generate a continuous-variable *W*-type entangled coherent state, described by Eq. (1), by using NVEs each located in a different cavity. Because of the long decoherence time of NVEs, the prepared *W* state can be stored for a long time. Note that NVEs have been recently considered as good memory elements in quantum information processing^39^,^40^,^42^,^45^,^46^,^47^,^48^,^49^,^51^.

As shown below, this proposal has the following features and advantages: (i) Different from the previous works^33^,^34^,^35^,^36^,^37^, the *W* state is prepared using NVEs (quantum memories) *instead of cavity photons*. Thus, the prepared *W* state can be stored for a long time due to the long decoherence time of the NVEs. (ii) Because cavity photons are virtually excited for most of the operation time, decoherence caused by the cavity decay and the unwanted inter-cavity cross talk is greatly suppressed. (iii) Each NVE is initially in the ground state. Thus, there is no need to initially prepare each NVE in a coherent state, which should greatly reduce its experimental difficulty. (iv) Moreover, only one external-cavity coupler qubit is needed, which simplifies the circuit. This method is quite general and can be applied to prepare the proposed *W* state with atomic ensembles or other spin ensembles based on cavity/circuit QED.

There are several additional motivations of this proposal:

- Planar superconducting TLRs with internal quality factors above one million (*Q* > 10^6^) have been recently reported^52^, for which the lifetime of microwave photons can reach ~1 ms. Comparably, a lifetime of ~1 s for an NVE has been experimentally reported^53^. Hence, a NVE is a good memory element for storing quantum states, superior to using cavity photons as memories.
- By local operation, the prepared *W* state of the NVEs can be mapped onto the cavities (see the “Quantum state transfer” subsection).
- The NVEs could be prepared in the ground state at a 40–50 mK or higher temperature^42^,^44^. The strong coupling of a superconducting qubit with a microwave resonator (e.g., *g*/2*π* ~ 360 MHz for a transmon qubit coupled to a TLR^54^,^55^) has been reported in experiments, and the strong coupling (~11 MHz) of an NVE to a TLR has recently been experimentally demonstrated^44^. Moreover, superconducting qubits, capacitively or inductively coupled to TLRs^13^,^56^,^57^,^58^,^59^,^60^,^61^,^62^,^63^,^64^,^65^,^66^,^67^,^68^, were previously employed for QIP. Hence, the model considered in this work is reasonable and physical.

Note that based on circuit QED, a number of proposals have been presented for creating entangled states (e.g., Bell states, NOON states, and GHZ states) of *microwave photons* distributed in different TLRs/cavities^57^,^58^,^60^,^63^,^65^,^67^. Instead of preparing entangled states of cavity microwave photons, this work focuses on preparing the NVEs in a *continuous-variable W*-type entangled coherent state.

In this work we will also discuss possible experimental implementation of our proposal and numerically calculate the operational fidelity for generating a *W*-type entangled coherent state of three NVEs. Our numerical simulation shows that highly-fidelity implementation of *W*-type entangled coherent states with three NVEs is feasible with rapid development of circuit QED technology. The numerical calculations in this work were performed using the QuTiP software^69^,^70^.

## Results

### *W*-state preparation

**Figure 1:**
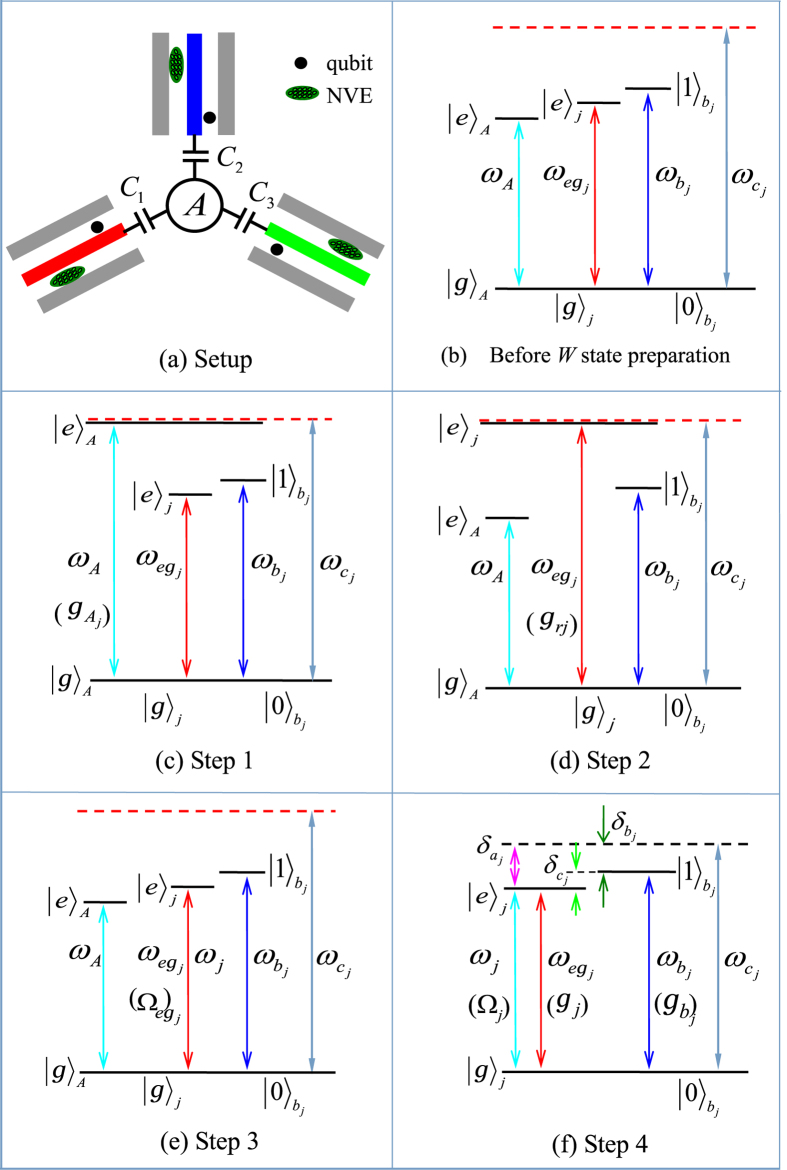
(**a**) Setup of the hybrid system consisting of a coupler qubit *A* and three cavities each hosting a qubit (a dark dot) and a nitrogen-vacancy center ensemble (a green oval). *C*_1_, *C*_2_ and *C*_3_ represent capacitors. An intracavity qubit can be an atom or a solid-state qubit. The coupler qubit *A* can be a quantum dot or a superconducting qubit. (**b**) Illustration of the decoupling among qubit *A*, cavity *j*, NVE *j* and qubit *j (j* = 1, 2, 3) before the *W*-state preparation. (**c**) The resonant interaction between qubit *A* and cavity *j* with coupling constant 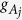 (used in step 1). (**d**) The resonant interaction between qubit *j* and cavity *j* with resonant coupling constant *g*_*rj*_ (used in step 2). (**e**) The resonant interaction between qubit *j* and the pulse with Rabi frequency 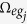 (applied for step 3). (**f**) The dispersive interaction between cavity *j* and qubit *j* with coupling constant *g*_*j*_ and detuning 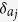, the dispersive interaction between cavity *j* and NVE *j* with coupling constant 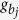 and detuning 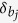, as well as the resonant interaction between qubit *j* and the pulse with Rabi frequency Ω_*j*_ (applied for step 4). Here, 
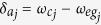
, with the transition frequency 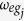 of qubit *j* and the frequency 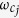 of cavity *j*, 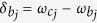 and 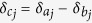, with 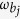 being the frequency of a bosonic mode describing NVE *j*. Since qubit *A* is not involved during the operation of step 4, qubit *A* is dropped off in (**f**) for simplicity. Note that in (**b**,**e**), the frequency of cavity *j* is highly detuned from those of qubit *A*, qubit *j* and NVE *j*, while in (**f**) the frequency of cavity *j* is adjusted such that cavity *j* is dispersively coupled to qubit *j* and NVE *j*. The bottom dark solid line in (**b**–**f**) also represents the ground state (i.e., the vacuum state) of cavity *j*.

Consider a hybrid system consisting of a coupler qubit *A* and three cavities, each hosting a qubit and an NVE [Fig. 1(a)]. Each cavity here is a one-dimensional transmission line resonator. The qubit and the NVE placed in cavity *j* are labelled as qubit *j* and NVE *j (j* = 1, 2, 3). The two levels of qubit *A* are denoted as |*g*〉_*A*_ and |*e*〉_*A*_, while those of qubit *j* as |*g*〉_*j*_ and |*e*〉_*j*_. The coupling and decoupling of each qubit from its cavity (cavities) can be achieved by prior adjustment of the qubit level spacings or the cavity frequency. For superconducting devices, their level spacings can be rapidly (within 1–3 ns^65^,^71^,^72^, (Yu, Y. & Han, S. private communication.)) adjusted by varying external control parameters (e.g., via changing the external magnetic flux threading the superconducting loop of phase, transmon, Xmon or flux qubits; see, e.g. refs 71 and 73, 74, 75, 76, 77, 78, 79, 80). In addition, as described in the Methods section, the coupling and decoupling of an NVE with a cavity can be made by rapidly adjusting the cavity frequency^81^,^82^.

Assume that the qubits, cavities, and NVEs are initially decoupled from one another [Fig. 1(b)]. The state-preparation procedure consists of four basic operations followed by a measurement on the state of each intra-cavity qubit, which is described below:

**Step 1.** Adjust the level spacings of the coupler qubit *A* so that it is resonantly coupled to each cavity [Fig. 1(c)]. Assume that the coupling constant of qubit *A* with cavity *j* is 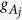. In the interaction picture, the Hamiltonian reads


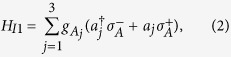


where 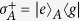 and 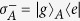 are the raising and lowering operators for qubit *A*, while *a*_*j*_ and 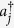 are the annihilation and creation operators for the mode of cavity *j (j* = 1, 2, 3). We set 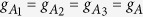, which can be met by a prior design of the sample with appropriate values of the coupling capacitance *C*_1_, *C*_2_, and *C*_3_. Assume now that qubit *A* is initially in the state |*e*〉_*A*_ and each cavity is initially in the vacuum state. It is easy to show that the state 
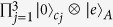
 of the system, under the Hamiltonian (2), evolves into


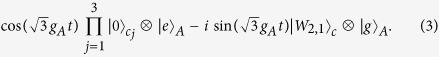


Here, the state |*W*_2,1_〉_*c*_ of the three cavities (1, 2, 3) is given by


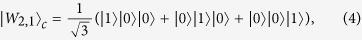


where |*i*〉| *j*〉|*k*〉 is the abbreviation of the state 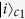

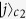

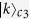
 of cavities (1, 2, 3) with *i*, *j*, *k* ∈ {0, 1}; |0〉 and |1〉 represent the vacuum state and the single-photon state, respectively. From Eq. (3), it can be seen that when the interaction time equals to 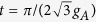, we can create the state |*W*_2,1_〉_*c*_ of the three cavities (1, 2, 3). Note that the coupler qubit *A* is in the ground state |*g*〉_*A*_ after the operation here and will remain in the ground state |*g*〉_*A*_ during the rest of the operations below.

**Step 2.** Adjust the level spacings of qubit *A* back to the original level structure such that it is decoupled from each cavity. In addition, adjust the level spacing of intra-cavity qubit *j* such that qubit *j* is resonantly coupled to cavity *j* [Fig. 1(d)]. The resonant coupling constant of qubit *j* with cavity *j* is denoted as *g*_*rj*_. In the interaction picture, the Hamiltonian can be written as


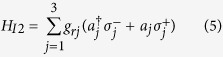


where 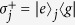 and 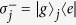 are the raising and lowering operators for qubit *j*. For simplicity, we set *g*_*r*1_ = *g*_*r*2_ = *g*_*r*3_ = *g*_*r*_, which can be achieved by tuning the level spacings of qubit *j* or adjusting the position of qubit *j* in cavity *j (j* = 1, 2, 3). It is easy to show that under this Hamiltonian (5), the time evolution of the state 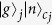 of qubit *j* and cavity *j* is described by


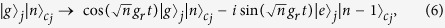


where 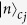 and 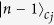 are the photon-number states of cavity *j*. Assume now that qubit *j* is initially in the state |*g*〉_*j*_. Choosing *t* = *π*/(2*g*_*r*_), one obtains the transformation 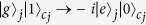. As a result, the state |*W*_2,1_〉_*c*_ of the three cavities turns into the following state of the three intracavity qubits (1, 2, 3)


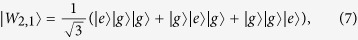


where |*i*〉| *j*〉|*k*〉 is the abbreviation of the state |*i*〉_1_| *j*〉_2_|*k*〉_3_ of intracavity qubits (1, 2, 3) with *i*, *j*, *k* ∈ {*g*, *e*}. It should be noted that each cavity returns to its original vacuum state after the operation here and will remain in the vacuum state during the following operations.

The condition *g*_*r*1_ = *g*_*r*2_ = *g*_*r*3_ = *g*_*r*_ is unnecessary. For the case of *g*_*r*1_ ≠ *g*_*r*2_ ≠ *g*_*r*3_, one can still obtain the state (7) from the state (4), by adjusting the level spacings of qubit *j* to bring qubit *j* on resonance with cavity *j* for a time *t*_*j*_ = *π*/(2*g*_*rj*_) (*j* = 1, 2, 3).

**Step 3.** Adjust the level spacings of intracavity qubits back to the original level configuration, such that they are decoupled from their cavities. Then apply a classical pulse to qubit *j*. The pulse is resonant with the |*g*〉_*j*_ ↔ |*e*〉_*j*_ transition of qubit *j* [Fig. 1(e)]. The interaction Hamiltonian in the interaction picture is given by


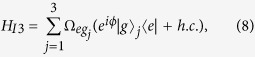


where 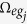 and *ϕ* are the Rabi frequency and the initial phase of the pulse, respectively. Set 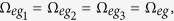 which can be readily met by adjusting the pulse intensities. It is easy to find that under the Hamiltonian (8), one can obtain the following rotations


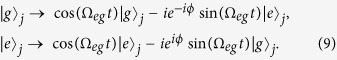


We set *t* = *π*/(4Ω_*eg*_) and *ϕ* = −*π*/2 to pump the state |*e*〉_*j*_ to |−〉_*j*_ and |*g*〉_*j*_ to |+〉_*j*_. Here, 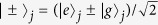 are the rotated basis states of qubit *j*. Thus, the state (7) becomes


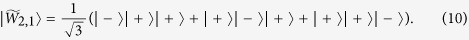


**Step 4.** Adjust the frequency of each cavity such that cavity *j* interacts with qubit *j* and NVE *j* [Fig. 1(f)]. Then apply a classical pulse (with frequency *ω*_*j*_ equal to 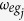) to qubit *j* [Fig. 1(f)]. Here, 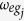 is the |*g*〉 ↔ |*e*〉 transition frequency of qubit *j*. The system Hamiltonian in the interaction picture yields


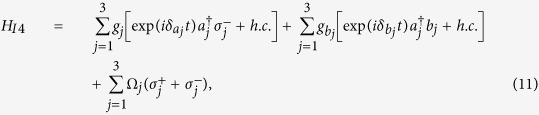


where 
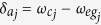
 and 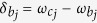 are the frequency detunings (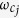 being the frequency of cavity *j* while 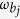 being the frequency of a bosonic mode describing NVE *j*), *b*_*j*_ is the bosonic operator for NVE *j*, *g*_*j*_ is the off-resonant coupling constant of qubit *j* with cavity *j*, 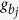 is the coupling constant of NVE *j* with cavity *j*, and Ω_*j*_ is the Rabi frequency of the pulse applied to qubit *j* [Fig. 1(f)]. Note that the second term of Eq. (11) describes three NVEs interacting with their respective cavities (see the Methods section). In a rotated basis {|+〉_*j*_, |−〉_*j*_}, one has 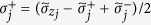 and 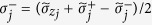, where 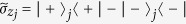, 
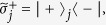
 and 
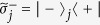
. Hence, the Hamiltonian (11) can be expressed as


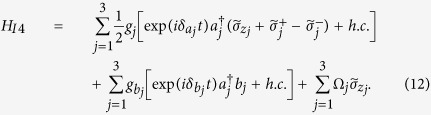


In a new interaction picture under the Hamiltonian 
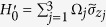
, one obtains from Eq. (12)


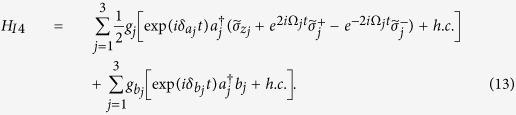


In the strong-driving regime 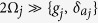, one can apply a rotating-wave approximation and eliminate the terms that oscillate with high frequencies. Thus, the Hamiltonian (13) becomes


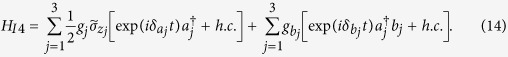


Consider now the large detuning conditions 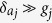 and 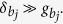 It is straightforward to show that the Hamiltonian (14) changes to (for details, see ref. 84)


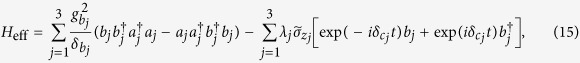


where 
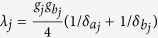
 and 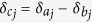. As mentioned previously, each cavity is in the vacuum state after the first three steps of operation above. In this case, the Hamiltonian (15) reduces to


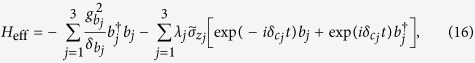


where the first term is the vacuum contribution Stark shift of NVEs, while the second term describes the coupling between qubit *j* and NVE *j*, mediated by the mode of cavity *j*. Because of using the large detuning technique, the effective coupling *λ*_*j*_ is smaller than *g*_*j*_ or 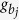 by at least one order of magnitude. Accordingly, the operation time for this last step of the operation (essentially based on a model via virtual transitions) would become longer by one order of magnitude, when compared with each of the first three steps of operation via resonant interaction.

In a new interaction picture under the Hamiltonian 
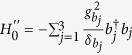
, the effective Hamiltonian (16) can be rewritten as


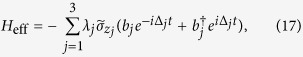


where 
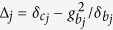
.

Let us now assume that the NVEs are initially in the state 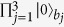. Thus, under the Hamiltonian (17), the joint state 
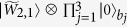
 of the three intracavity qubits and the three NVEs evolves into


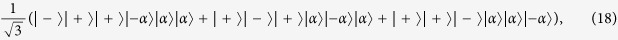


with


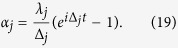


Here, 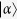 (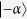) is a coherent state and we have set *α*_1_ = *α*_2_ = *α*_3_ = *α* for simplicity (which can be met for identical qubits, NVEs, and cavities). After returning to the original interaction picture by performing a unitary transformation 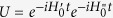, the state (18) becomes


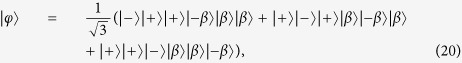


where a common phase factor is discarded, 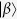 (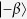) is a coherent state, and


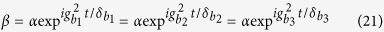


for


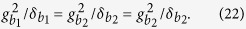


The condition (21) is automatically satisfied for identical NVEs and cavities. The state (20) can be expressed as


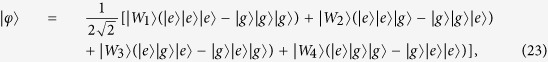


where |*W*_1_〉, |*W*_2_〉, |*W*_3_〉 and |*W*_4_〉 are the macroscopic *W*-type entangled coherent states of three NVEs, given by


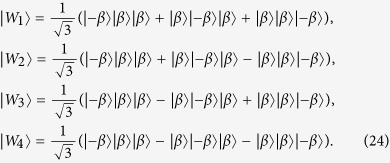


Now a measurement is separately performed on each intra-cavity qubit along a measurement basis {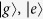}. If qubits (1, 2, 3) are measured in the state (i) |*e*〉|*e*〉|*e*〉 or |*g*〉|*g*〉|*g*〉, (ii) |*e*〉|*e*〉|*g*〉 or |*g*〉|*g*〉|*e*〉, (iii) |*e*〉|*g*〉|*g*〉 or |*g*〉|*e*〉|*e*〉, and (iv) |*e*〉|*g*〉|*g*〉 or |*g*〉|*e*〉|*e*〉, one can see from Eq. (23) that the three NVEs are respectively prepared in the *W* states |*W*_1_〉, |*W*_2_〉, |*W*_3_〉 and |*W*_4_〉, respectively.

**Figure 2:**
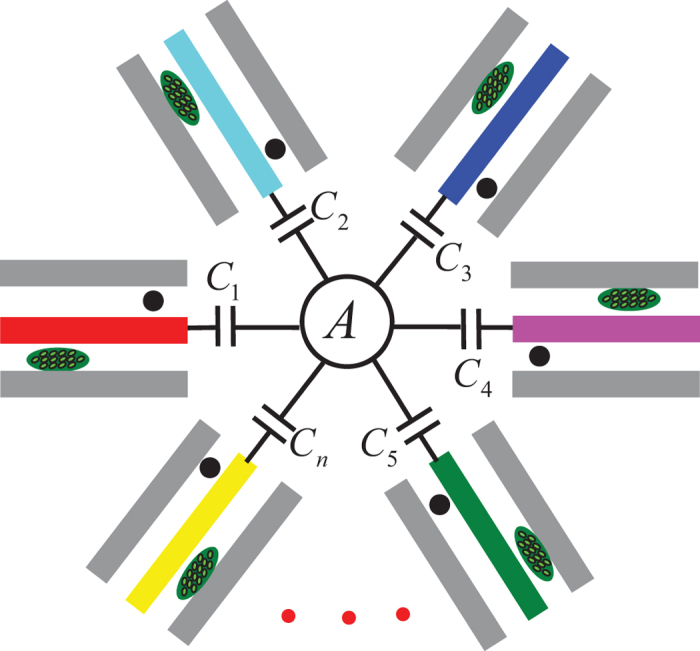
Diagram of a coupler qubit *A* and *n* cavities each hosting a qubit (a dark dot) and a NVE (a green oval). Qubit *A* is capacitively coupled to each cavity.

This method can be extended to a more general case. Consider a hybrid system composed of *n* cavities, each hosting a qubit *j* and an NVE *j (j* = 1, 2 ···*n*) and connected to a coulper qubit *A*, as shown in Fig. 2. Assume that the initial state of the system is 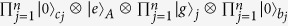. Employing the four-step procedure described above, it is straightforward to show that the *n* NVEs can be prepared in a *W*-type entangled coherent state. Let *m*_*j*_ = 0 represent qubit *j* being measured in the state |*g*〉, while *m*_*j*_ = 1 indicates qubit *j* being measured in the state |*e*〉. If the *n* intracavity qubits are measured in the state |*m*_1_*m*_2_ ···*m*_*n*_〉, the *n* NVEs will be prepared in the macroscopic *W*-type entangled coherent state


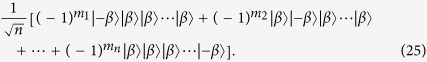


Before ending this section, several points need to be addressed as follows:

- From the description given above, one can see that only resonant interactions are used for the first three steps of operation, which can thus be completed within a very short time (e.g., by increasing the pulse Rabi frequencies and the qubit-cavity coupling constants). In contrast, the last step of operation employs a large detuning, leading to a relatively long operation time. However, cavity photons were virtually excited during this step of operation. Hence, in the present proposal each cavity remains in a vacuum state for most of the operation time.
- Coupling/decoupling the NVE with the cavity in step 4 requires dynamic tuning of the cavity frequency^81^,^82^. It is known that tuning the cavity frequency (e.g., via the insertion of the flux-tunable inductor in the resonator) can significantly reduce the quality factor of the cavity, which would decrease the fidelity of the prepared *W* state. Alternatively, to have the cavities coupled with or decoupled from the NVEs, one can choose to adjust the level spacings of the NVEs (e.g., by varying the external magnetic fields applied to the NVEs^48^,^85^). However, it is an experimental challenge to change the NVE level spacings quickly. Typically, it takes more than 1 ms to adjust the NVE level spacings (Saito, S. *private communication*.), which would significantly prolong the entire operation time and thus the operation fidelity would be expected to be low due to decoherence. Therefore, we choose using the cavity of adjustable frequency in this paper.
- As shown above, the intracavity-qubit *W* state of Eq. (7) can be produced within a very short time, because the first two steps of operation, for producing this intracavity-qubit *W* state (7), employ resonant interactions. Alternatively, this intracavity-qubit *W* state (7) can be prepared via a detuned interaction between the coupler qubit *A* and each cavity^13^,^64^,^68^. Thus, there are no cavity photons excited during the entire state preparation. However, the time required for preparing the *W* state (7) becomes much longer due to the use of a detuned interaction, and thus decoherence from the qubits may pose a significant problem.
- Placing a qubit in each cavity [Fig. 1(a)] is necessary in view of energy conservation. During the last step, each cavity remains in a vacuum state and thus there is no energy transfer from each cavity onto the NVEs. Note that the intracavity qubits are the ones that absorb energy from the pulses applied to them and then transfer their energy to the NVEs through interaction with the NVEs. Thus, in spite of initially being in the ground state, the NVEs can be prepared in a *W*-type entangled coherent state.
- As discussed previously, a measurement of the states of each intra-cavity qubit is needed during preparation of the *W*-class entangled coherent states. To the best of our knowledge, all existing proposals for creating entangled coherent states of two components 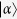 and 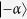 based on cavity QED or circuit QED require a measurement on the states of auxiliary qubits or qutrits^63^,^85^,^86^,^87^,^88^,^89^,^90^,^91^,^92^,^93^,^94^.

### Possible experimental implementation

Superconducting qubits play important roles in quantum information processing^73^,^75^,^76^,^95^,^96^,^97^. In addition, circuit QED is a realization of the physics of cavity QED with superconducting qubits or other solid-state devices coupled to a microwave cavity on a chip and has been considered as one of the most promising candidates for quantum information processing^75^,^76^,^95^,^96^,^97^,^98^,^99^,^100^. Above, we considered a general type of qubit for both the intracavity qubits and the coupler qubit. As an example of experimental implementation, let us now consider each qubit as a superconducting transmon qubit.

The dynamics of the lossy system, with finite qubit relaxation and dephasing and photon lifetime included, is determined by the following master equation


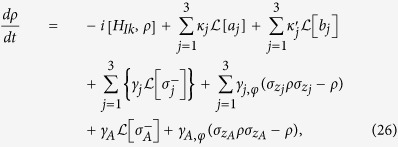


where *H*_*Ik*_ is either *H*_*I*1_, *H*_*I*2_, *H*_*I*3_, or *H*_*I*4_; *j* represents qubit *j (j* = 1, 2, 3); 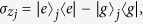 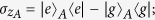 and 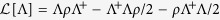, with 
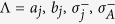
. In addition, *κ*_*j*_ is the decay rate of cavity *j*, 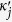 is that of NVE *j*, *γ*_*j*_ (*γ*_*A*_) is the energy relaxation rate of the level 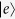 of qubit *j (A*), and *γ*_*j*,*φ*_ (*γ*_*A*,*φ*_) is the dephasing rate of the level 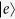 of qubit *j (A*).

The fidelity of the operation is given by^101^


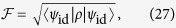


where 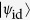 is the output state of an ideal system (i.e., without dissipation and dephasing), while *ρ* is the output-state density operator of the system when the operations are performed in a realistic physical system.

We now numerically calculate the fidelity of operation. Since the first three steps employ resonant interactions, we will look at the operational fidelity for each of these steps to see how short one should make the typical operation time for each step to combat decoherence while still being able to generate the entanglement with high fidelity. For simplicity, we will consider the ideal output state of the previous step of operation as the input state of the next step of operation when we analyze the operational fidelities for the first three steps. In addition, we will investigate the fidelity for the entire operation, which will be calculated by numerically solving the master equation with the initial state of the whole system as an input, but without making any approximation. Without loss of generality and for simplicity, we will consider identical transmon qubits, cavities, and NVEs. In this case, we have *g*_*Aj*_ = *g*_*A*_, *g*_*rj*_ ≡ *g*_*r*_, *g*_*j*_ ≡ *g*, and 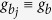 (*j* = 1, 2, 3). We set 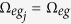 and Ω_*j*_ = Ω (*j* = 1, 2, 3). The decoherence times of transmon qubits and NVEs used in the numerical simulation are: 
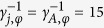
 *μ*s, 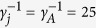 *μ*s (which is a conservative estimate compared with those reported in experiments^102^,^103^,^104^). In addition, we choose 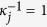 *μ*s and 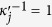 ms in the numerical simulation (*j* = 1, 2, 3).

### A. Fidelity for the first three steps

**Figure 3:**
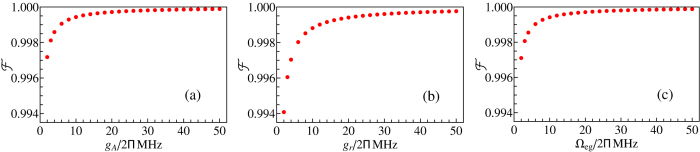
(**a**) Fidelity for step 1. (**b**) Fidelity for step 2. (**c**) Fidelity for step 3.

The operation fidelities are plotted in Fig. 3(a–c), which are for step 1, step 2, and step 3, respectively. Figure 3 shows that the fidelity for step 1, step 2, or step 3 increases drastically with *g*_*A*_, *g*_*r*_, or Ω_*eg*_ and reaches a high value 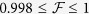 for *g*_*A*_/(2*π*), *g*_*r*_/(2*π*), Ω_*eg*_/(2*π*) ∈ [5 MHz, 50 MHz], which corresponds to the operation time ~3–30 ns. The analysis given here demonstrates that in order to combat decoherence while obtain the entanglement with a high fidelity ~1, one should make the typical operation time within a few nanoseconds for each of the first three steps, and a high fidelity ≥0.998 can be achieved even by increasing the operation time to ~30 ns.

### B. Fidelity for the entire operation

**Figure 4:**
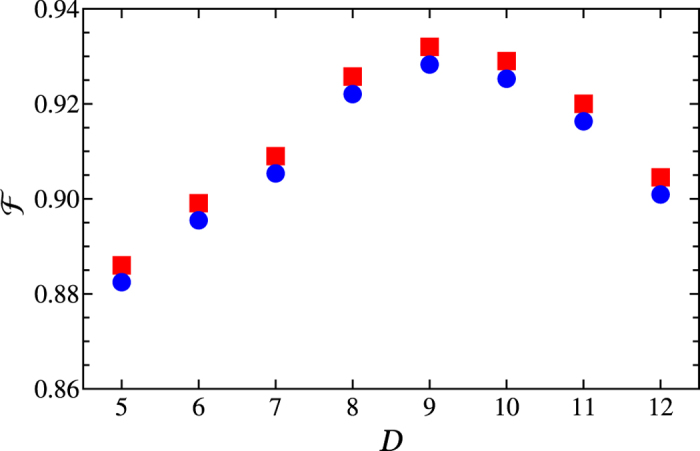
Fidelity 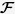 versus reduced detuning 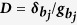. The red squares correspond to the case without considering the errors and decoherence for the first three-step operation, while the blue dots correspond to the case after the errors and decoherence for the first three-step operation are taken into account. The parameters used here are described in the text.

The fidelity for the entire operation is calculated based on Eq. (27), where the ideal output state is 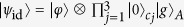 [with 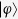 given by Eq. (20) or Eq. (23)] and *ρ* is obtained by numerically solving the master equation (26) for an initial input state 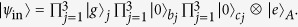 We choose *g*_*A*_/(2*π*) = 50 MHz, *g*_*r*_/(2*π*) = *g*/(2*π*) = 5 MHz, and *g*_*b*_/(2*π*) ~ 4 MHz^44^. We here select *g*_*r*_ = *g* because the resonant coupling constant *g*_*r*_ and the off-resonant coupling constant *g* are both the same order of magnitude for superconducting qubits. Other parameters used in the numerical simulation are: Ω_*eg*_/(2*π*) = 50 MHz, Ω/(2*π*) = 100 MHz (available in experiments^105^,^106^), and 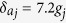 (obtained by numerically optimizing the system parameters). With the choice of these parameters, the fidelity versus 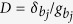 is plotted in Fig. 4, which demonstrates that for *D* ~ 9, a high fidelity ~93.2% can be achieved for the state 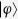 with |*β*| = 1.2. For *D* ~ 9, the entire operation time is estimated to be ~1.14 *μ*s, much shorter than the decoherence times of transmon qubits and NVEs used in our numerical simulation but a little longer than the cavity decay time. Figure 4 also shows that the fidelity heavily depends on *D* (or the detuning 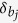). The fidelity reaches its maximum as *D* increases to 9. However, it drops down when *D* becomes larger than 9. This means that further increasing the detuning 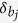 will have an adverse effect on the fidelity. The interpretation for this is: As the detuning 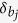 becomes larger than the optimum value 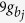 (2*π* × 36 MHz) (i.e., the value where the large detuning is well satisfied), the NVE-cavity coupling becomes weaker, which increases the operation time and thus the effect of decoherence from transmon qubits and NVEs on the fidelity becomes more apparent.

**Figure 5:**
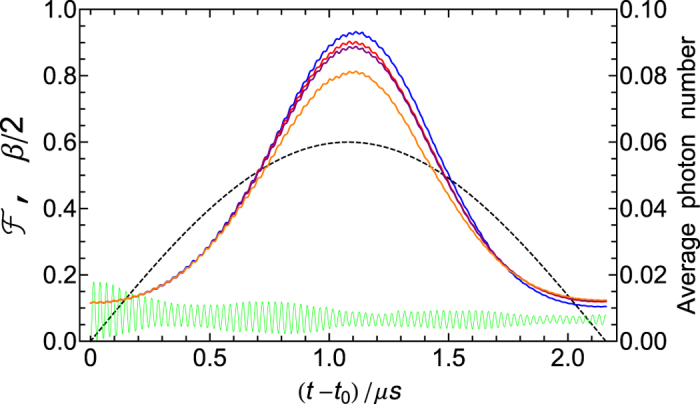
(**a**) The operational fidelity 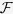, the amplitude |*β*| (or |−*β*|), and the photon number of each cavity versus *t* − *t*_0_ (i.e., the time required for the last step of operation), for the case of considering the identical NVE-cavity coupling strengths and NVE-cavity frequency detunings. The blue, black, and green curves are plotted for reduced detuning 
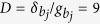
 and parameters used in Fig. 4. The blue curve represents the operational fidelity, which is calculated for an ideal state 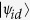 (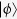) with |*β*| = 1.2. For *t* − *t*_0_ = 1.08 *μ*s (i.e., the time required for preparing the state 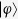 with |*β*| = 1.2 during the last step of operation), the fidelity 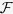 reaches the maximum ~93.2%. The black curve represents the value of |*β*|/2 or |−*β*|/2. The green curve indicates the photon number (enlarged 10 times) of each cavity. (**b**) The operation fidelity versus *t* − *t*_0_ for inhomogeneous NVE-resonator couplings and unequal NVE-resonator frequency detunings. The brown, purple, and red curves represent the operational fidelities for NVE spin coherence times *t* = 10 *μ*s, 100*μ*s, and 1 ms, respectively. The maximum fidelities (corresponding to the peak values of the brown, purple, and red curves) are, respectively, 81.5%, 89.0%, and 90.3%.

Note that although the entire operation time is longer than the cavity decay time used in our numerical simulations, the effect of the cavity decay on the fidelity is negligible. This is because: the first three steps are completed within a very short time due to using the resonant interaction, and (as illustrated in Fig. 5) the number of photons occupied in each cavity during the last step of operation is quite low due to using a large-detuning technique. Indeed, to reduce decoherence from the cavity decay, one can employ a longer cavity-decay time in the numerical simulation, which however would require cavities with a higher-*Q* quality factor and thus may pose a challenge in experiments.

Figure 5 is plotted by choosing the detuning *D* = 9 and using the same parameters for Fig. 4. For simplicity, Fig. 5 only shows the curves corresponding to the operation time *t* − *t*_0_ required for the last step of operation. Here, *t* is the entire operation time while *t*_0_ is the time required for the first three steps of operation. For the values of *g*_*A*_, *g*_*r*_, and Ω_*eg*_ chosen above, *t*_0_ is ~36 ns. The blue curve represents the fidelity, which is calculated for an ideal state 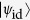 (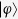) with |*β*| = 1.2. The black curve represents the value of |*β*|/2 or |−*β*|/2. The green curve indicates the average photon number for each cavity. The blue curve indicates that the fidelity increases when *t* − *t*_0_ approaches 1.08 *μ*s (which is the time required for the last step of operation for preparing the desired state 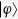 with |*β*| = 1.2). The maximum fidelity depicted by the blue curve of Fig. 5 is in good agreement with that shown in Fig. 4 for *D* = 9. In addition, the green curve shows that the average number of photons excited in each cavity is less than 0.02, implying that the cavity photons are almost not excited during the last step of operation.

In a realistic situation, it may be a challenge to obtain identical NVE-resonator frequency detunings and homogeneous NVE-resonator coupling strengths. Thus, we numerically calculate the fidelity by setting 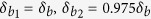 and 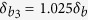, 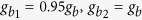 and 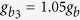. As shown in the brown, purple, and red curves of Fig. 5, one can see that a high fidelity 81.5%, 89.0%, 90.3% can be obtained for the NVE spin coherence times 10 *μ*s, 100 *μ*s, and 1 ms, respectively.

According to experimental reports^81^,^82^, the cavity frequency can be rapidly adjusted by Δ*ω*_*c*_/(2*π*) = 500~740 MHz. As a conservative consideration, for Δ*ω*_*c*_/(2*π*) = 500 MHz, the detuning 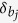 changes to 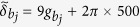 MHz, which can be further written as, e.g., 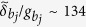 for the identical NVE-cavity coupling strengths *g*_*bj*_/(2*π*) ≡ *g*_*b*_/(2*π*) = 4 MHz chosen above. This result shows that the decoupling of the cavities with the NVEs, which was required during the *W*-state preparation, can be well met by adjusting the cavity frequency. As discussed previously, the coupling or decoupling of the qubits with the cavities can be readily made by adjusting the level spacings of the qubits.

*T*_1_ (energy relaxation time) and *T*_2_ (dephasing time) can be made to be on the order of 20–80 *μ*s for state-of-the-art superconducting transmon devices^102^,^103^,^104^. The typical transition frequency of a transmon qubit is between 2 and 10 GHz^77^,^107^. As an example, consider each cavity of frequency *ν*_*c*_ ~ 5 GHz. Hence, for the 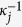 used in the numerical calculation, the required quality factor of each cavity is *Q*_*j*_ ~ 3.1 × 10^4^, which is accessible in experiments because a quality factor *Q* ~ 5 × 10^4^ for CPW resonators with loaded NVEs has been experimentally demonstrated^44^. The analysis given here shows that a high-fidelity implementation of the three-NVE *W*-type entangled coherent state 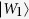, 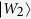, 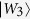, or 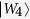 described by Eq. (24) is feasible with rapid development of circuit QED techniques.

### Quantum state transfer

Consider a cavity and an NVE inside the cavity. Based on Eq. (35) (see the Methods section), the NVE-cavity interaction Hamiltonian can be written as


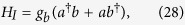


where we set *δ* = *ω*_*b*_ − *ω*_*c*_ = 0. Assume now that the initial state of the cavity and the NVE is given by |0〉_*c*_⊗|*β*〉_NVE_, where |0〉_*c*_ is the vacuum state of the cavity while |*β*〉_NVE_ is the coherent state of the NVE, given by 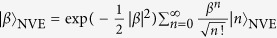. In terms of 
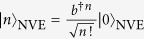
, one can describe the system initial state as


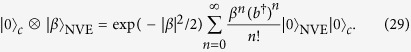


Making use of the Hamiltonian (28), we can obtain the transformations 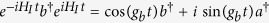. For *g*_*b*_*t* = *π*/2, one has 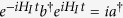. Under the Hamiltonian (28) and after an evolution time *t* = *π*/(2*g*_*b*_), the state of the system can be written as


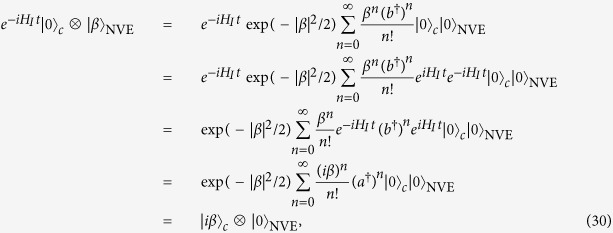


where we have used 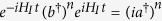 and 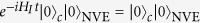.

In the same manner, after an evolution time *t* = *π*/2*g*_*b*_, the state 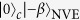 of the cavity and the NVE is transformed to 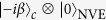. Given the above results, one can transfer a macroscopic *W*-type entangled coherent state from the NVEs into the cavities. For instance, the above state 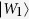 of the three NVEs is transferred onto the three cavities, becoming


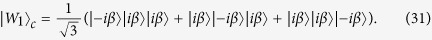


## Discussion

We should mention that in 2015, Song *et al*. proposed a scheme to generate a GHZ-type macroscopic entangled coherent state of NVEs that are coupled to a superconducting flux qubit^85^. In contrast, we here proposed a protocol for creating a macroscopic *W*-type ECS with NVEs coupled to different cavities. Hence, compared with^85^, our proposal is for a different system and it differs in both the prepared states and the coupling structure.

A method has been presented to generate a *continuous-variable W*-type entangled coherent state of NVEs in circuit QED. As shown above, this proposal offers some distinguishing features and advantages. The prepared *W* state of NVEs can be mapped onto the cavities by local operations. Our numerical simulations show that the high-fidelity implementation of *W*-type entangled coherent states with three NVEs is feasible with rapid development of circuit QED technology.

## Methods

### NVE-cavity interaction Hamiltonian

**Figure 6:**
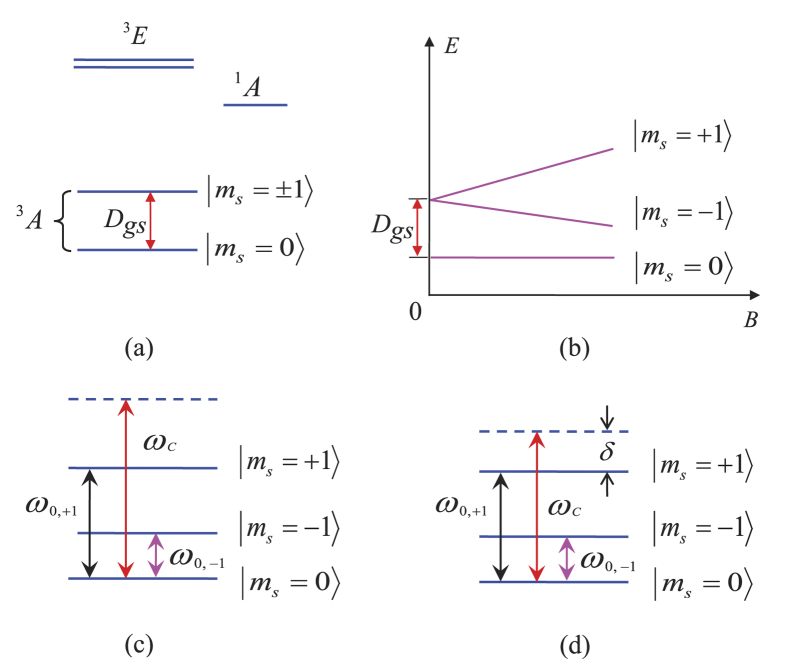
(**a**) Schematic diagram of electronic and spin energy levels of a nitrogen-vacancy center. (**b**) The ground electronic-spin levels of an NV center in the presence of an external magnetic field parallel to the crystalline axis. Here *B* and *E* represent the magnetic field and energy, respectively. (**c**) Illustration of the cavity decoupled from the NV center. Here, *ω*_*c*_ is the cavity frequency, while *ω*_0,−1_ (*ω*_0,+1_) is the energy gap between the |*m*_*s*_ = 0〉 and |*m*_*s*_ = −1〉 (|*m*_*s*_ = +1〉) levels of the NV center. The cavity frequency *ω*_*c*_ is sufficiently larger than *ω*_0,+1_ and *ω*_0,−1_, such that the cavity mode is highly detuned (decoupled) from both the |*m*_*s*_ = 0〉 ↔ |*m*_*s*_ = −1〉 transition and the |*m*_*s*_ = 0〉 ↔ |*m*_*s*_ = +1〉 transition. (**d**) Illustration of the cavity being coupled to the |*m*_*s*_ = 0〉 ↔ |*m*_*s*_ = +1〉 transition with a detuning *δ* = *ω*_*c*_ − *ω*_0,+1_, but decoupled from the |*m*_*s*_ = 0〉 ↔ |*m*_*s*_ = −1〉 transition of the NV center.

As shown in Fig. 6(a), the energy levels of an NV center consist of a ground state ^3^*A*, an excited state ^3^*E* and a metastable state ^1^*A*. Both ^3^*A* and ^3^*E* are spin triplet states while the metastable ^1^*A* is a spin singlet state^108^,^109^. The NV center has an *S* = 1 ground state with zero-field splitting *D*_*gs*_/(2*π*) = 2.88 GHz between the |*m*_*s*_ = 0〉 and |*m*_*s*_ = ±1〉 levels [Fig. 6(a)]. By applying an external magnetic field along the crystalline axis of the NV center^48^,^84^, an additional Zeeman splitting between |*m*_*s*_ = ±1〉 sublevels occurs [Fig. 6(b)].

If we need to eliminate the coupling of the cavity with the NV center, one can adjust the cavity frequency *ω*_*c*_ to have *ω*_*c*_ sufficiently larger than *ω*_0,+1_ and *ω*_0,−1_, such that the cavity mode is highly detuned (decoupled) from both the |*m*_*s*_ = 0〉 ↔ |*m*_*s*_ = −1〉 transition and the |*m*_*s*_ = 0〉 ↔ |*m*_*s*_ = +1〉 transition [Fig. 6(c)]. Here, *ω*_0,+1_ (*ω*_0,−1_) is the transition frequency between the two levels |*m*_*s*_ = 0〉 and |*m*_*s*_ = +1〉 (|*m*_*s*_ = −1〉). On the other hand, one can adjust the cavity frequency such that the cavity mode is coupled with the transition between the ground level |*m*_*s*_ = 0〉 and the excited level |*m*_*s*_ = +1〉, but still decoupled from the transition between the two levels |*m*_*s*_ = 0〉 and |*m*_*s*_ = −1〉 [Fig. 6(d)]. Note that for a superconducting transmission line resonator, the rapid tuning of cavity frequencies by a few hundred MHz in 1–2 nanoseconds has been demonstrated in experiments^81^,^82^). During the *W*-state preparation described in the Results section, we assume that the level splitting of the NV center is fixed.

An NV center is usually treated as a spin while an ensemble of NV centers is treated as a spin ensemble (i.e., an NVE). Let an NVE be placed at an antinode of a single mode of the electromagnetic field. When the cavity is coupled to the |*m*_*s*_ = 0〉 ↔ |*m*_*s*_ = +1〉 transition, but decoupled from the |*m*_*s*_ = 0〉 ↔ |*m*_*s*_ = −1〉 transition [Fig. 6(d)], the system Hamiltonian in the interaction picture reads (in units of *ħ* = 1)


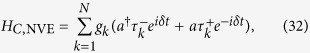


where *δ* = *ω*_*c*_ − *ω*_0,+1_, *ω*_*c*_ is the eigenfrequency of the cavity mode, *a* (*a*^†^) is the corresponding annihilation (creation) operator of the cavity mode, 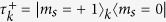 and 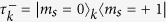 are the raising and lowering operators for the *k*th spin, and *g*_*k*_ is the coupling strength between the cavity and the *k*th spin. We then define a collective operator


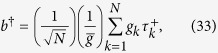


with 
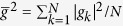
, and 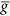 is the root mean square of the individual couplings.

Under the condition of a large *N* and a very small number of excited spins (compared to the number *N*), *b*^†^ behaves as a bosonic operator and the spin ensemble behaves as a bosonic mode. Thus, we have [*b*, *b*^†^] ≈ 1, and *b*^†^*b*|*n*〉_*b*_ = *n*|*n*〉_*b*_^48^,^110^, where


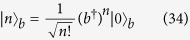


with |0〉_*b*_ = |*m*_*s*_ = 0〉_1_|*m*_*s*_ = 0〉_2_ ···|*m*_*s*_ = 0〉_*N*_. It is easy to verify that the frequency *ω*_*b*_ of the bosonic mode describing the NVE is equal to the transition frequency *ω*_0,+1_ between the ground level |*m*_*s*_ = 0〉 and the excited level |*m*_*s*_ = +1〉 of each spin (i.e. *ω*_*b*_ = *ω*_0,+1_). For simplicity we have defined |*m*_*s*_ = +1〉 = |+1〉 and |*m*_*s*_ = 0〉 = |0〉.

Therefore, the Hamiltonian (32) can be further rewritten as


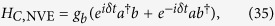


with 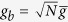. Based on Eq. (35), one can find that for the case of three NVEs each placed in a cavity, the Hamiltonian for the three NVEs interacting with their respective cavities would be the second term of Eq. (11).

### NVE-cavity coupling selection

During the last step of the *W* state preparation, we would require the coupling of each cavity with the |*m*_*s*_ = 0〉 ↔ |*m*_*s*_ = +1〉 transition while decoupling each cavity from the |*m*_*s*_ = 0〉 ↔ |*m*_*s*_ = −1〉 transition. The advantage of this is that the created *W* state has a mode frequency equal to *ω*_0,+1_, which is adjustable by varying the magnetic field applied to the NVEs [Fig. 6(c,d)]. Instead of using the coupling of each cavity with the |*m*_*s*_ = 0〉 ↔ |*m*_*s*_ = +1〉 transition, one can employ the coupling of each cavity with the |*m*_*s*_ = 0〉 ↔ |*m*_*s*_ = ±1〉 transition (i.e., the transition between the ground state |*m*_*s*_ = 0〉 and the degenerate excited states |*m*_*s*_ = ±1〉). However, there is an inevitable shortcoming, i.e., the created *W* state has a fixed mode frequency, which is equal to *ω*_0,±1_ = 2*π* × 2.88 GHz [Fig. 6(a)] and thus cannot be adjusted.

## Additional Information

**How to cite this article**: Liu, T. *et al*. Generation of a macroscopic entangled coherent state using quantum memories in circuit QED. *Sci. Rep*. **6**, 32004; doi: 10.1038/srep32004 (2016).
